# Quantitative Dynamic Allodynograph—A Standardized Measure for Testing Dynamic Mechanical Allodynia in Chronic Limb Pain

**DOI:** 10.3390/s23187949

**Published:** 2023-09-18

**Authors:** Noy Turgeman Dahan, Jean-Jacques Vatine, Irit Weissman-Fogel, Hana Karpin, Sharon Shmuely, Tami Bar-Shalita

**Affiliations:** 1 Department of Occupational Therapy, School of Health Professions, Faculty of Medicine, Tel Aviv University, Tel Aviv-Yafo 69978, Israel; noy.turgdahan@gmail.com; 2 Reuth Rehabilitation Hospital, Tel Aviv-Yafo 6772830, Israel; hanakarpin@gmail.com (H.K.); sharone.kutas@gmail.com (S.S.); 3 Faculty of Medicine, Tel Aviv University, Tel Aviv-Yafo 69978, Israel; vatinejj@gmail.com; 4 Physical Therapy Department, Faculty of Social Welfare and Health Sciences, University of Haifa, Haifa 3103301, Israel; ifogel@univ.haifa.ac.il

**Keywords:** mechanical allodynia, central sensitization, clinical pain

## Abstract

Background: Dynamic mechanical allodynia (DMA) is both a symptom and a central sensitization sign, yet no standardized method for quantifying the DMA area has been reported. This study aimed to establish psychometric properties for *Quantitative Dynamic Allodynography* (*QDA*), a newly developed protocol measuring the DMA area as a percentage of the body surface. Methods: Seventy-eight patients aged 18–65 diagnosed with chronic complex regional pain syndrome (CRPS) participated in this study. Test–retest reliability was conducted twice, one week apart (N = 20), and inter-rater (N = 3) reliability was conducted on 10 participants. Disease severity (*CRPS Severity Score*, *CSS*), pain intensity (VAS), and quality of life (SF-36) measures were utilized to test construct validity. Results: High inter-rater reliability (intraclass correlation coefficient (ICC) = 0.96, *p* < 0.001) and test–retest reliability (*r* = 0.98, *p* < 0.001) were found. Furthermore, the QDA score was found to be correlated with the CSS (*r* = 0.47, *p* < 0.001), VAS (*r* = 0.37, *p* < 0.001), and the SF-36 physical health total (*r* = −0.47, *p* < 0.001) scores. Conclusion: The QDA is the first developed reliable and valid protocol for measuring DMA in a clinical setting and may be used as a diagnostic and prognostic measure in clinics and in research, advancing the pain precision medicine approach.

## 1. Introduction 

Dynamic mechanical allodynia (DMA) is a type of pain provoked by stroking movements on the skin which normally do not provoke pain [1]: Following an injury, the barrage of noxious information carried by the C—nociceptive neurons sensitize the second-order neurons at the dorsal horn of the spinal cord, a phenomenon named central sensitization. As a result, non-noxious stimuli carried by A-beta neurons are perceived as painful [1,2]. Clinically, DMA appears within (the primary hyperalgesia area) and around (the secondary hyperalgesia area of) the injured area [1], where expansion of the DMA area indicates a more extensive central sensitization [3,4]. Therefore, the measurement of the DMA surface can be used as a marker of central sensitization magnitude [5]. Thus, being both a symptom and a sign, it is crucial to assess DMA. 

Complex regional pain syndrome (CRPS), a chronic pain condition usually affecting a single limb, is manifested in motor, trophic, autonomic, and sensory abnormalities [6]. DMA is a common component [7] of the sensory disturbances required to be evaluated in the diagnosis of CRPS [8,9], and its presence is associated with poor syndrome prognosis [10,11]. Moreover, DMA is a major cause of decreased function and health-related quality of life [12]. In a recent report aiming to identifying a set of CRPS core clinical outcomes, the need for an essential clinical outcome that maps the body area corresponding to each patient’s allodynia using an allodynia map was agreed upon [6]. Thus, it is essential not only to indicate DMAs presence and intensity but also to quantify its surface using a valid and reliable method.

Currently DMA is measured by brushing the area reported by the patient to be sensitive utilizing tools and techniques which are not standardized for DMA measurement, thus, yielding variation in the force, distance, and the speed applied [13], which may compromise the measurement of outcomes. While a method for measuring static mechanical allodynia was reported [14,15], to our knowledge there is no standardized valid and reliable method for measuring DMA. To bridge this gap, we recently developed Quantitative Dynamic Allodynograph (QDA) as a non-invasive indicator of the presence, extent, and magnitude of the DMA. This is a six-stage protocol for measuring DMA area using a scientific image-analysis software and calculating the area as a percentage of the body surface. This protocol is anchored in the method for testing static allodynia [15,16] and the Lund and Browder atlas [17] (see the QDA description under Methods). This study aimed to establish psychometric properties for the newly developed QDA. Specifically, the research goals were twofold: (1) To measure the convergent validity of the QDA through its correlation with CRPS severity index, clinical pain, and a physical health-related quality of life questionnaire, and (2) to test its test–retest and inter-rater reliability. The quantification of the DMA phenomenon via the establishment of QDA psychometric properties could ameliorate not only the evaluation of patients suffering from DMA but also reveal the extent of central sensitization, crucial for promoting the development of successful treatment plans which are tailored to the individual. 

## 2. Methods 

This was a cross-sectional study, approved by the Institutional Review Board (2017-14). All participants signed an informed consent form before enrolling in the study.

### 2.1. Participants 

The sample size required for correlation analysis (first goal) was based on the G* power 3 software and calculated a priori [18], with an effect size f² = 0.30, α = 0.05, and power (1-β err prob) = 0.80. The calculation yielded a sample size of n = 64. 

Seventy-eight patients with CRPS in the upper or lower limb, aged 18–65 years, diagnosed according to the Clinical Budapest Criteria [19] by a specialist physician in physical medicine and rehabilitation, and in pain management, participated in this study. The Clinical Budapest Criteria consist of continuous pain that is disproportionate to any inciting event and a report of at least one symptom in at least three of the following categories (sensory, vasomotor, sudomotor, and motor/trophic). Further, at least one sign had to occur in 2 or more of the categories (sensory, vasomotor, sudomotor, and motor/trophic), with no other condition accounting for these signs and symptoms. The Clinical Budapest Criteria have a sensitivity of 0.99 and a specificity of 0.68 for the diagnosis of CRPS [19]. Participants were recruited in consecutive sampling from the Center for Rehabilitation of Pain Syndromes, Reuth Rehabilitation Hospital. Inclusion criteria stipulated chronic CRPS—4 months or more after receiving the diagnosis, without language barriers. Exclusion criteria included other chronic pain diagnosis; current or history of any psychiatric, neurodevelopmental, or neurological disorder; uncorrected visual or auditory impairment; and pregnancy.

### 2.2. Measures

**Figure 1:**
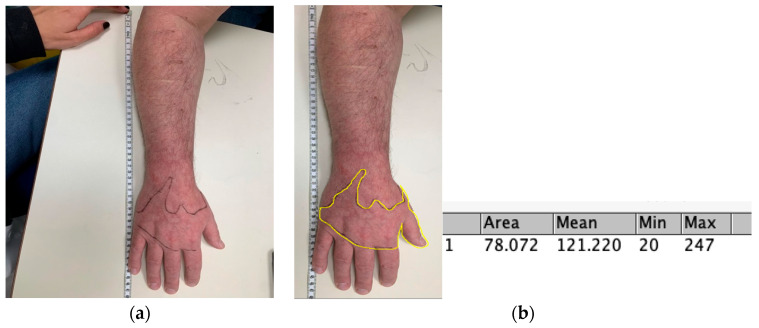
(**a**) The Quantitative Dynamic Allodynograph (QDA) stages iii + iv. The marked border indicates the allodynic area according to patient report. (**b**) The Quantitative Dynamic Allodynograph—stage v: Electronically marking and calculating the DMA area utilizing ImageJ, which output is in cm^2^. The Mean, Min and Max are calculated from the values of the pixels along the line. Specifically, Mean—smooths the current image by replacing each pixel with the proximity mean. Minimum (Min)—this filter performs grayscale erosion by replacing each pixel in the image with the smallest pixel value in that pixel’s proximity. Maximum (Max)—this filter performs grayscale dilation by replacing each pixel in the image with the largest pixel value in that pixel’s proximity.

*The Quantitative Dynamic Allodynograph* (*QDA*) is a protocol for measuring the dynamic mechanical allodynic area as a percentage of the body surface, anchored in the method for testing static allodynia [15,16], however, combining manual measurement and computerized image-analysis processing. The QDA is conducted in six stages: (i) *Identifying the sensitive area* based on verbal questioning and subject responses, i.e., “Where does it hurt?”. (ii) *Defining the skin area indicating a change in sensation* (hypersensitivity/poor sensation) using a cotton swab (Applicator 6” wooden stick 1 end cotton, Sion Medical Ltd.) 15 cm long in a horizontal grip, with pressure < 2 gr in four directions, proximal, distal, medial, and finally, lateral directions, in relation to the allodynic area. This was carried out until the area was defined and marked by points using a safe pencil on the body surface (“Stop me when you feel a change in sensation”). (iii) *Defining the allodynic area*—using cotton swabbing, touching every point marked in the previous step and asking whether the touch provoked pain > 3/10 in numerical pain scale (NPS). When the touch is <3, the examiner continues to move distally or proximally until the subject rates the pain caused by the touch as 3/10 or higher, followed by framing the area using a safe pencil by connecting the points marked on the body (Figure 1). (iv) *Scanning the area* using a measuring tape near and parallel to the affected limb’s length while the subject is lying on their back and stomach. In each position, the limb and the measuring tape are scanned together using a smartphone (Figure 1a). (v) *Calculating the allodynic area* by uploading the scans to the ImageJ software, a Java-based image processing program developed at the National Institutes of Health and the Laboratory for Optical and Computing Instruments [20], which automatically calculates the marked area (Figure 1b)*. (vi) *Calculating the area as a percentage of the whole body surface* using the Lund and Browder [17] atlas indicating the percentage of body area relative to the whole body surface, a well-known and accepted index in the field of burns. This percentage, relative to the whole-body area, forms the QDA score (i.e., higher values indicate larger percentage of body surface), based on the following formula: 

Allodynia area (%) = allodynia area measured via the ImageJ software (cm^2^) ÷ area of whole body part measured via ImageJ software (cm^2^) × standard percentages of the whole body part according to the Lund and Browder method [17]. 

* ImageJ, a low-cost scientific image-analysis software supporting standard image processing functions. It is a public domain Java image processing and analysis program, running either as a downloadable application or an online applet, requiring Java 1.5 or a more recent virtual machine. It can calculate pixels and selected area values, measure distances and angles, create line profile plots and geometric transformations, such as scaling. Spatial calibration providing real world dimensional measurements (i.e., millimeters) is available [21].

*The CRPS Severity Score (CSS)* [19], a standard index administered by a physician and based on the diagnostic criteria of CRPS (the Budapest Criteria), aimed to provide a quantitative assessment of CRPS severity and monitor its changes over time. The index includes 8 symptoms and 8 signs. Each sign/symptom is counted as a point and added to a total score ranging from 0–16, where higher scores indicate higher syndrome severity [8,9,22]. In this study the CSS was used to test construct (convergent) validity. 

*The Short-form McGill Pain Questionnaire (SF-MPQ)* [23,24] is a standard self-report questionnaire comprising two parts. The first consists of 15 pain descriptors (11 with sensory and 4 with emotional-valence) aiming to provide different dimensions of the pain experience (sensory and affective), as well as the overall intensity of the pain experience. Pain intensity is measured according to a 4-point scale (0—no pain; 1—mild; 2—moderate; 3—severe). The total score of this part ranges from 0 to 45, where higher scores represent increased pain experience. The second part includes a measurement of current pain intensity using (i) the Visual Analogue Scale (VAS) a 10 cm long horizontal line with anchors ranging from ‘no pain’ to ‘maximal pain’ and (ii) Present Pain Intensity—verbal bar with 5 degrees (0—no pain; 1—very weak pain; 2—weak pain; 3—moderate pain; 4—fairly strong pain; 5—very strong pain). In this study, the SF-MPQ was used to test construct convergent validity. 

*The Short-form health survey Questionnaire (SF-36)* [25,26], a standard reliable and valid shortened version of the MOS—Medical outcomes study [25]—aimed to evaluate health-related quality of life. The SF-36 includes 36 items divided into eight scales: physical functioning, emotional functioning, social functioning, energy level, personal well-being, pain, vitality, and general health, together eliciting two summary indices, *Mental health* and *Physical health*. The response scales vary in items and ranges from 2 to 6 points. In this study, the SF-36 was used to test construct (convergent) validity using the *Physical health* index.

*Demographic information* -self-report questionnaire developed for this study comprising demographic data (e.g., disease duration, marital status, education).

### 2.3. Procedure

The study took place at Reuth Rehabilitation Hospital (Tel-Aviv, Israel), from June 2017 to August 2021. Eligible patients were invited to see a physician, pain specialist, where they were diagnosed with CRPS using the Clinical Budapest criteria and evaluated to determine CRPS severity using the CSS. Thereafter, the study was carried out in two phases: The first phase designed to establish the tool reliability by evaluating *Test–retest reliability,* conducted twice, one week apart, by the same assessor, and *Inter-rater reliability* performed by three different trained assessors, each testing the QDA on different days within one week, at the same time of day. The second phase, designed to establish *construct-convergent validity* was composed of conducting the QDA test, performed by one of the researchers (NT), and the self-report questionnaires, completed by participants in another research session. All research sessions were performed within one week and conducted in a quiet room with an ambient temp of 24 °C. 

### 2.4. Statistical Analysis 

Statistical analyses were performed using SPSS^®^ V26 (IBM Corp., Armonk, NY, USA). Data was aggregated with descriptive statistics by data type. Normal distribution of the dependent variables was tested using the Shapiro Willks test, and variables were analyzed accordingly. We used descriptive statistics to describe the study population. The intraclass correlation coefficients (ICCs) test was used to test reliability between testers [low ICC < 0.50; moderate ICC = 0.50–0.75; high ICC = 0.75–0.90; excellent ICC > 0.90] [27]. The Pearson/Spearman correlation coefficient test was used to test test–retest reliability (tested by one assessor), as well as correlations between study measures, to test construct validity. A significance level of *p* < 0.05 was set for all tests.

## 3. Results

### 3.1. Participants 

Patients with CRPS, age range 18–65, participated in the study: In the first phase, designed to establish the reliability of the tool, 30 subjects participated (n = 10 for testing inter-rater reliability and n = 20 for testing test–retest reliability), among whom 13 also participated in the second phase. In the second phase, designed to establish the validity of the tool, 61 subjects participated (see Table 1). 

### 3.2. Phase I—Establishing Inter-Rater Reliability and Test–Retest Reliability for the QDA

**Figure 2:**
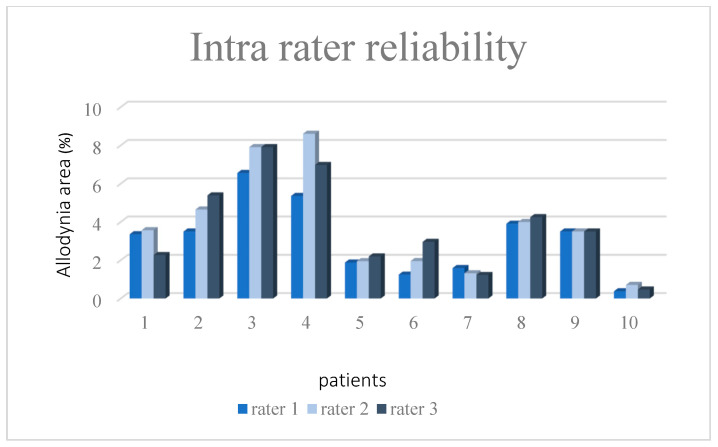
QDA Inter-rater reliability.

*Inter-rater reliability*, tested using three assessors, each assessing 10 participants, found excellent intraclass correlation coefficient (ICC) = 0.96, *p* < 0.001, see Figure 2. Further, testing *test–retest reliability* in 20 participants found very high correlation between the two scores of each participant (*r* = 0.98, *p* < 0.001), demonstrating excellent *test–retest reliability.*

Three raters assessed DMA in ten patients with CRPS, demonstrating high inter-rater agreement on the QDA scores, namely, the DMA area as a percentage of the total body surface. The intraclass correlation coefficient (ICC) = 0.96, *p* < 0.001. *QDA—Quantitative Dynamic Allodynograph*; *DMA—dynamic mechanical allodynia*.

### 3.3. Phase II-Convergent Validity for the QDA

Table 2 presents the descriptive statistics according to the type of distribution found for each independent variable.

Statistically significant moderate correlations were found between the QDA score (area of allodynia percentage) and the CSS, as well as the SF-36 Physical health summary score. Statistically significant low–moderate positive correlation was found between the QDA score (area of allodynia percentage) and the MPQ pain rating score. In addition, a statistically significant low positive correlation was found between emotional aspects in the MPQ questionnaire and the QDA score. Table 3 presents the correlation found between the QDA score and study measures used to test the validity, demonstrating that these properties are related yet differ from the QDA. 

## 4. Discussion

The aim of the present study was to establish psychometric properties (reliability and validity) for the QDA among subjects with CRPS. Findings indicate that the QDA is a reliable and valid protocol for measuring the DMA area as a percentage of the body surface. 

### 4.1. Reliability of the QDA 

Reliability is concerned with the reproducibility and consistency of a measurement method, assessing the extent to which the method is free of random error [28], requiring values above 0.90 in tools evaluating individuals [29]. In the present study, high inter-rater reliability was found for the QDA (ICC = 0.97), ensuring a stable, rater-independent measure, thus indicating a standardized protocol. In a study which established psychometric properties for the static mechanical allodynograph and testing patients with CRPS, similar inter-rater reliability was reported (ICC = 0.97), which the researchers concluded as an excellent inter-rater reliability [14].

In the present study, we also found an excellent test–retest reliability for the QDA (*r* = 0.98), indicating that DMA is a stable and measurable phenomenon. This finding is particularly important since pain is conceived as a subjective phenomenon [30], where accordingly pain patients report feeling their credibility is being questioned by caregivers, family, and medical staff [31]. Moreover, the pain intensity that patients are routinely required to report may be impacted by psychological factors and personal beliefs leading to ratings that are not necessarily the actual sensory sensation of pain. Therefore, an accurate measure for the sensory dimension of pain is crucial for the management and treatment of pain [5,30,32]. Thus, this study presents excellent reliability for the QDA, indicating that pain sensing can be reliably measured.

### 4.2. Construct Validity of the QDA

The validity of a measure is the extent that it measures the phenomenon it purports to measure [33,34]. Utilizing a convergent method for testing construct validity assumes that the measure being tested has relationships with variables, indicating they share a somewhat similar content [35,36,37]. In the current study, we asked participants to answer the following question: “On a scale of 1–10, does it hurt you 3 or more?” This question refers to the sensory dimension, and therefore, we hypothesized that we would find relationships with other indices related to the sensory dimension of pain and its manifestation (i.e., CSS, pain level according to VAS in the MPQ questionnaire and a summary score of physical health in the SF-36 questionnaire), indicating convergent validity. 

This study found a moderate correlation between the QDA score and pain intensity level measured via the MPQ-VAS, establishing convergent validity for the QDA. While in the QDA protocol, the subject is asked to rate pain following the administration of a non-painful stimulus, in the MPQ, the subject is asked to rate spontaneous current pain (not evoked) using the VAS. Backonja and Stacey [38] argue that spontaneous pain and mechanical allodynia are mediated by different mechanisms. Moreover, examining pain mechanisms in the oral mucosa after dental treatment in rats, Ito et al. [39] found that spontaneous pain is mediated by Transient Receptor Potential Channels (TRP)V1 and TRPA1, while mechanical allodynia is mediated by TRPV4 [39]. Further, testing the effect of Huperzine A (extracted from the Chinese club-moss plant used in Chinese medicine to reduce inflammation) [40] on spontaneous pain and mechanical allodynia in mice, it was found that Huperzine A attenuated mechanical allodynia, while it had no effect on spontaneous pain [40]. On the contrary, Gotttup et al. [41] studied the effect of lidocaine (sodium channel blocker [42]) and ketamine (antagonist for the N-methyl-D-aspartate receptor [42]) in reducing spontaneous pain and mechanical allodynia, demonstrating that spontaneous pain and mechanical allodynia were reduced by ketamine, implying a common central mechanism. Indeed, both phenomena are derived from sensitization of second order nociceptive neurons resulting in spontaneous firing and neural responses to innocuous stimuli [43], which behaviorally manifested as spontaneous pain and mechanical allodynia, respectively. Thus, common and distinct mechanisms underlying spontaneous pain and mechanical allodynia may explain the moderate association that we found between QDA and MPQ-VAS [43,44]. 

Correlation was also found between the QDA and CSS score, which measures CRPS severity. The CSS includes patient report and physician examination evaluating allodynia as part of the sensory criteria [8,9,45,46], as well as the motor and autonomic symptoms and signs [19]. Indeed, finding a distinct medium relationship between CSS and the QDA indicates the impact of the sensory domain on the overall CSS score and thus supports the QDA as a measure of the pain sensory domain. 

Further, a significant moderate negative correlation was found between the SF-36 summary score of physical health, examining health related quality of life, and the QDA score. Previous reports indicate that patients with CRPS experience disrupted quality of life [12,47,48,49,50], which was associated with higher pain ratings using the VAS [51] and higher symptom severity evaluated via the CSS [51]. However, the relationship between allodynia and quality of life has not yet been directly studied among CRPS patients though DMA negatively affects almost every dimension of daily life. For example, patients who suffer from allodynia feel pain while showering, dressing, walking outside, or just feeling the wind on their limbs. Consequently, to decrease their pain, they tend to reduce activity and participation levels, which in turn negatively affect their physical health. Applying the QDA enables assessing the link between DMA and health related quality of life. To the best of our knowledge, this is the first study investigating the relationship between allodynia and physical health-related quality of life among CRPS patients, reinforcing the direct link between the sensory aspect of pain and physical health-related quality of life. 

## 5. Conclusions

In this study psychometric properties for the newly developed QDA were established among individuals with CRPS. The research results provide convergent construct validity, inter-rater reliability, and test–retest reliability for the QDA. This QDA protocol may be used as a diagnostic and prognostic measure among CRPS patients, as well as other chronic pain patients who suffer from DMA. Furthermore, it may be used as a goal directed outcome measure, indicating rehabilitation efficiency, and advancing the pain precision medicine approach. 

## Figures and Tables

**Table 1:** Participant demographics and characteristics.

		First Phase N = 30 Mean (SD)	Second Phase N = 61 Mean (SD)
Age		32.02 (11.87)	35.61 (12.09)
Years of education			13.05 (2.19)
Years from injury		1.66 (2.65)	1.97 (2.41)
		N (%)	N (%)
Gender	Men	18 (58.1)	29 (47.5)
	Women	12 (41.9)	32 (52.5)
Type of CRPS	1 2	14 (45.2) 16 (51.6)	38 (62.35) 23 (37.65)

SD—standard deviation.

**Table 2:** Descriptive statistics of the research variables—Phase II (Mean, SD Median, and IQR).

Measures	Median (IQR)
QDA	5.03 (1.25–6.72)
CSS	12 (10–14)
MPQ-VAS	7 (5–8)
MPQ Affect total	6 (3–10)
	Mean (SD)
SF-36 Physical Health sum score	27.17 (13.37)

QDA—Quantitative Dynamic Allodynograph; CSS—CRPS Severity Score, possible score range 0–16; MPQ—McGill pain Questionnaire, possible score range 4–16; VAS—Visual Analogue Scale, possible score range 0–10; SF-36—Short-form health survey, possible score range 0–100; SD—standard deviation; IQR—Interquartile range.

**Table 3:** Correlations between the QDA and CSS, MPQ, and SF-36-Physical Health total scores (N = 61).

	QDA *r*	*p*
CSS	0.47	<0.001
MPQ-VAS	0.37	<0.001
MPQ Affect total	0.26	0.026
SF-36 Physical Health total score	−0.47	<0.001

QDA—Quantitative Dynamic Allodynograph; CSS—CRPS Severity Score; MPQ—McGill pain Questionnaire; VAS—Visual Analogue Scale; SF-36—Short-form health survey Questionnaire.

## Data Availability

Dataset can be made available upon request.
